# Why does parental language input style predict child language development? A twin study of gene–environment correlation

**DOI:** 10.1016/j.jcomdis.2015.07.004

**Published:** 2015

**Authors:** Philip S. Dale, Maria Grazia Tosto, Marianna E. Hayiou-Thomas, Robert Plomin

**Affiliations:** a Department of Speech & Hearing Sciences, University of New Mexico, United States; b Department of Psychology, Tomsk State University, Russia; c Department of Psychology, University of York, United Kingdom; d Social, Genetic and Developmental Psychiatry Research Centre, King's College London, United Kingdom

**Keywords:** Child language, Input, Genetics

## Abstract

There are well-established correlations between parental input style and child language development, which have typically been interpreted as evidence that the input style causes, or influences the rate of, changes in child language. We present evidence from a large twin study (TEDS; 8395 pairs for this report) that there are also likely to be both child-to-parent effects and shared genetic effects on parent and child. Self-reported parental language style at child age 3 and age 4 was aggregated into an ‘informal language stimulation’ factor and a ‘corrective feedback’ factor at each age; the former was positively correlated with child language concurrently and longitudinally at 3, 4, and 4.5 years, whereas the latter was weakly and negatively correlated. Both parental input factors were moderately heritable, as was child language. Longitudinal bivariate analysis showed that the correlation between the language stimulation factor and child language was significantly and moderately due to shared genes. There is some suggestive evidence from longitudinal phenotypic analysis that the prediction from parental language stimulation to child language includes both evocative and passive gene–environment correlation, with the latter playing a larger role.

**Learning outcomes**: The reader will understand why correlations between parental language and rate of child language are by themselves ambiguous, and how twin studies can clarify the relationship. The reader will also understand that, based on the present study, at least two aspects of parental language style – informal language stimulation and corrective feedback – have substantial genetic influence, and that for informal language stimulation, a substantial portion of the prediction to child language represents the effect of shared genes on both parent and child. It will also be appreciated that these basic research findings do not imply that parental language input style is unimportant or that interventions cannot be effective.

## Highlights

• Parental input–child language correlations are ambiguous with respect to cause.
• Measures of parental language input style show substantial genetic influence.
• Shared genes between parents and children explain some of the observed correlation.
• Passive GE correlation may be stronger than evocative GE correlation.

## 1 Introduction

Since the seminal work of Snow (1972), an extensive body of research has been conducted on the characteristics of speech directed to, or produced in interaction with, young children in the early stages of language acquisition, typically below three years of age (see Hoff, 2006; Rowe, 2012, for reviews of this work). A central question has been the extent to which these features of child-directed speech (CDS) are predictive of children's rate of language development. In recent years, a primary focus of attention has been on quantity of input. Although numerous earlier studies found a positive relationship between quantity of input and rate of language development, this result was made more vivid to a wider population by the work of Hart and Risley (1995), who documented wide variations in the amount of speech to young children, and the extent to which these were reflected in vocabulary differences in the children. But beyond the quantitative differences, numerous qualitative differences have also been shown to predict child language both concurrently and longitudinally, generally with weak-to-moderate effect sizes. These qualitative, facilitating aspects of CDS are found in all the subdomains of language (phonology, lexicon, syntax, semantics, and pragmatics), and include greater vocabulary diversity, appropriately scaffolded mean length of utterance (MLU), increased repetition, exaggerated prosody, promotion of joint attention, proportion of conversation-eliciting speech as opposed to behavior regulation, semantic contingency, decontextualized language use such as narratives, ‘grammatical tutorials’ such as sentence recasts and expansions, and others (Hoff, 2006; Rowe, 2012).

These features of CDS are not all equally important at various stages of development. Promotion of joint attention and use of exaggerated intonation, are likely to be most important in the earliest stages of development, while vocabulary diversity and use of sentence recasts are likely to be more important later. A very clear example of this shift was demonstrated in Rowe's (2012) study of predictors of vocabulary growth between 18 and 42 months. Quantity of parental input was most important during the second year of life, while diversity of parental vocabulary was more important in the 3rd year, and the use of decontextualized language such as narratives and explanations was most important in the 4th year. The present study focuses on a broad dimension of CDS which combines quantity and quality, namely the tendency to incorporate talk with children into everyday routines, as well as a second dimension, the potential tendency to provide corrective feedback.

Most of the identified facilitative features of CDS are intercorrelated, and also correlated with socioeconomic status (SES), usually assessed as parental education. Nevertheless, Rowe (2012) and others have found that measures of specific features of CDS, representing the proximal environment, add significantly to the prediction of child language beyond the prediction from the distal variable of SES. In part this reflects the fact that within levels of SES, there is great variability in parental behavior. More broadly, environmental influences on language development are extensive and widely varied, including, for example, prenatal care and nutrition, exposure to toxins and disease, caregiver education and mental health, type and quality of childcare, and multilingual vs. monolingual context (see other papers in this special issue for some current examples). In many cases, it is likely that the effect of these environmental variables is mediated at least in part by parental language input.

An important qualification to this summary is that it is based primarily on research in literate, technological, primarily Caucasian Western societies. There is also much research that parent–child verbal interaction patterns are quite different in traditional, nonliterate societies, and also (in different ways) in East Asian cultures (Johnston & Wong, 2002; Ochs & Schieffelin, 1984). Although not as specifically focused on parental language as the preceding research, research in African-American communities on parent–child interaction and its predictive significance for language development has shown that both the meaning and predictive effects of specific behaviors, e.g., sensitivity, and negative-intrusive behaviors, can vary by racial group (Pungello, Iruka, Dotterer, Mills-Koonce, & Reznick, 2009; see also Dudley-Marling & Lucas, 2009, for an alternative, critical perspective on Hart & Risley). There is considerable debate at present about the extent to which different patterns of verbal interaction can support language development (Lieven, 1994; Hoff, 2006), but that is outside the scope of this paper.

### 1.1 Interpreting phenotypic correlations

The body of research confirming correlations between aspects of parental language input and child language outcomes is extensive and impressive in the consistency of the findings (Hoff, 2006; Rowe, 2012). The most typical interpretation is a causal one: these features of the input facilitate language acquisition. Based on that interpretation, it is natural to translate positive correlations into recommendations for parents, educators, and clinicians (e.g., Finestack & Fey, 2013; Girolametto, Weitzman, & Earle, 2013). In this paper we focus on an inherent ambiguity in such correlations, which comes from the fact that most of this research is based on biologically related parents and children, who share both genes and environments.

An alternative explanation for the correlations between parental input and child language is that children influence parents as much or more than parents influence children. Children who are more talkative, more advanced, and/or talk in specific ways may elicit parental speech (or the speech of other adults) with the hypothesized facilitating features to a greater extent than children who are less talkative, less advanced, or talk in different ways (Bohannon & Marquis, 1977; DeThorne & Chanell, 2007; Paul & Elwood, 1991). Even if the child language outcome is manifested later in development than the measured parental behavior, those outcomes may represent a stable aspect of their behavior, and the earlier behavior may have influenced parental input. Another alternative, familiar to anyone who has taken a basic statistics class, is the possibility that both measured variables are influenced by an unmeasured ‘third variable’. An especially plausible potential candidate for that third variable is the shared genetic endowment of parent and child. Parents who adopt the style of CDS discussed above may be generally more sensitive to language, and pass that on to their children genetically. Indeed, the first alternative explanation may be due to the second, when the features of children that influence parents are themselves at least in part due to shared genes. There is, in fact, considerable evidence for a genetic influence on children's rate of language development, small but significant at age 2, and generally rising across development to adolescence (Hayiou-Thomas, Dale, & Plomin, 2012; Spinath, Dale, Price, & Plomin, 2004).

Both child-to-parent effects and shared genetic effects lead to *gene–environment correlation*, a correlation between the environment that children experience and their genetic endowment. Cases where children influence parents are called in behavioral genetics *evocative gene–environment correlation*, because the correlation is the result of children evoking a particular kind of parental language response. When a ‘third variable’ of shared genes influences both parent and child directly, this is *passive gene–environment correlation*, because the correlation between the child's genes and his/her environment does not require any active response on the part of either the child or the parent.

**Fig. 1:**
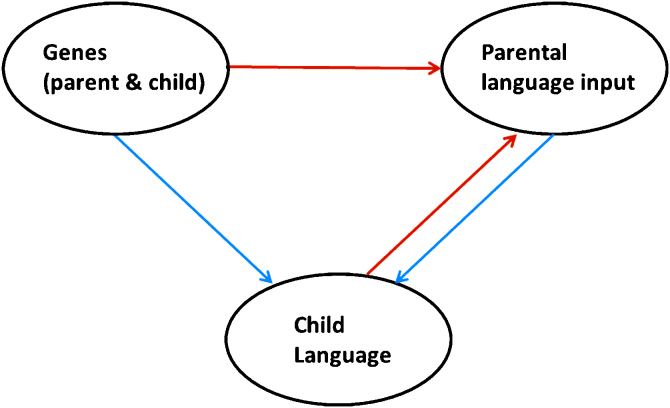
The interplay of genetic and environmental factors, particularly parental language input, in language development. Causal influences depicted as lighter red links contribute to gene–environment correlation.

These three possibilities, the ‘standard causal’ one and the two alternatives, are of course not mutually exclusive. To the extent that each may play a role, a more comprehensive view of the likely role of genes and environment is summarized in Fig. 1.

### 1.2 Identifying and measuring gene–environment correlation as an explanation for phenotypic correlations

An example from the development of literacy can clarify this research approach, in particular, how gene–environment correlations are detected. In the preschool years, there has been much emphasis on early literacy experience as a facilitative factor for emergent and early literacy. Oliver, Dale, and Plomin (2005) examined the information provided by parents on their children's preliteracy knowledge (letter, word and rhyme knowledge at 4), early literacy experience (book-reading activities, number of children's books, etc.) and reading ability (teacher rating) at the end of the second year of school. Both preliteracy knowledge and early literacy experience were predictors of reading ability (*r* = .29 and .19, respectively). Not surprisingly, both preliteracy knowledge and reading ability showed genetic influence as shown by the discrepancy between the intraclass correlations (ICCs; closely related to the familiar Pearson correlation) between twins in monozygotic (MZ) and dizygotic (DZ) twin pairs. But so did early literacy experience, a nominally environmental variable, with genetic variance accounting for nearly a quarter of the observed variance (*h*^2^ = .22). The evidence for this gene–environment correlation is that the intraclass correlation within twin pairs for early literacy experience was also higher for MZ pairs, who have identical DNA sequences, than for DZ pairs, who have 50% similar sequences on average.^1^ In other words, MZ twin pairs experienced more similar environments than DZ twin pairs; because the parents and other family factors were the same in both kinds of twin pairs, the only possible explanation is the genetic difference between the two kinds of twin pairs. In the second stage, the extent to which the gene–environment correlation is responsible for the observed phenotypic correlation was determined. This influence is measured by *bivariate heritability*, the proportion of the phenotypic correlation which is due to genes influencing both the environmental variable and the child outcome. It too is estimated by comparing correlations for MZ twin pairs with those for DZ pairs, but in this case the correlations are between the environmental measure and the child outcome (Plomin, DeFries, Knopik, & Neiderhiser, 2013). In this case, the genetic contribution was small, but significant; the bivariate heritability estimate of .05 was just over one-quarter of the phenotypic correlation.

Although the twin method has been used to examine gene–environment correlation for many aspects of cognitive, academic, and socioemotional development (cf. Plomin et al., 2013), its application to the study of early first language development faces two related major challenges. Child-specific measures of the environment are essential, not just family-level measures, and large samples of twins are needed. The most common environmental measures for language development (e.g., MLU, semantic contingency) are derived from the transcription and analysis of language samples, which is a highly time- and labor-intensive process. Few projects can undertake obtaining and analyzing two language samples for each of hundreds, preferably thousands, of families. To our knowledge, only a single twin study of child language input and development based on language-sample derived measures of the input has been conducted (DeThorne & Hart, 2009). A total of 207 twin pairs (mean age 7 years) from the Western Reserve Reading Project (Petrill, Deater-Deckard, Thompson, DeThorne, & Schatschneider, 2006) participated. Each twin in a pair participated in a conversation with a separate, unrelated examiner during play with modeling clay. The use of unrelated examiners made it possible to focus specifically on evocative gene–environment correlation. Although substantial genetic effects on all conversational measures – talkativeness, mean length of utterance, vocabulary diversity, and grammatical complexity – were found, this was not true for examiner language. That is, there was no evidence that the examiners were modifying their language in response to the child, and therefore none for evocative gene–environment correlation, so there was no need to conduct the second phase of the analysis. However, this was a single study, conducted at a specific, later developmental period than early language development. In addition, it was not focused on parental input, which is often the largest and also the most studied source of language input in early development.

Another potential source of information on the language environment is parental self-report. Although parent report has been widely used as a measure of child language development, its use to measure the input is not, and may seem quite radical. This method, however, is not uncommon in the socioemotional domain. For example, Parent et al. (2014) found that parental reports of negative parenting behavior (though not positive parenting) were valid in terms of correlation with observations. Similarly, Plomin, Riess, Hetherington, and Howe (1994) found that parents’ rating of their own positivity and negativity toward their adolescent children gave similar results to those provided by the adolescents themselves.

### 1.3 The present study

In the present study, we took advantage of information in the Twins Early Development Study (TEDS) on child language at 3, 4, and (for a subsample) 4.5 years, and also on reported parent language input style at 3 and 4 years for a very large number of families. The genetically sensitive and longitudinal design of TEDS makes it possible to address the question in the title of this paper in two ways: first, by decomposing the correlation between parent language input and child language into genetic and environmental factors; and second, by comparing the cross-correlation from parent language input to later child language with that from child language to later parent language input.

Our specific research questions were: first, do these parent input measures predict child language at the same age and later? Second, is there evidence for gene–environment correlation in the form of a genetic influence on the parent input measures? Third, what proportion of the phenotypic correlation between parental input and child language is due to the genetic factors shared by parent and child? (These questions are addressed through the first, behavioral genetic method described above.) And fourth, although the twin design does not itself enable discrimination of evocative vs. passive gene–environment correlation, is there phenotypic evidence for directionality of effect? (This is addressed through the second, longitudinal phenotypic method.)

## 2 Method

### 2.1 Participants

The Twins Early Development Study (TEDS) is a very large, longitudinal population-representative study of twins born in 1994–1996 in England and Wales. Families of twins were identified by the UK Office of National Statistics from birth records and were contacted when the children were 1 year old. Of all families (*n* = 16,810) who responded that they were interested in participating in TEDS, over 12,000 families have been involved in TEDS since its inception, at least for one assessment point. Given the size of the sample, parent report served as the major measurement method in the preschool years. TEDS has remained reasonably representative of UK census data with respect to percent of white families, parental educational qualifications, and percent of maternal employment, especially during the early years which are the focus of the present study (Haworth, Davis, & Plomin, 2013).

Zygosity of twin pairs was determined using parent questionnaires of physical similarity administered when the children were 18 months, 3 years, and 4 years; DNA testing was conducted when zygosity was not clear from physical similarity or the parents requested it (see Kovas, Haworth, Dale, & Plomin, 2007, for details on this determination). Prior to analysis, the following exclusion criteria were applied: specific medical syndromes such as Down syndrome and other chromosomal abnormalities, cystic fibrosis, and cerebral palsy; severe hearing loss; autism spectrum disorder; organic brain damage; extreme outliers for birthweight and gestational age; heavy maternal alcohol consumption during pregnancy; and intensive care after birth. In addition, only families in which English was the primary home language were included in the present study.

These criteria resulted in a total sample size of 8395 pairs, 2819 monozygous (MZ) pairs, 2842 same-sex dizygotic (DZ) pairs, and 2734 opposite-sex DZ pairs. These totals include all individuals who contributed at least one data point. In addition to the main TEDS assessment which was done at ages 3 and 4, a subset of TEDS twins was also given an in-depth, in-home assessment of verbal and nonverbal ability at 4.5 years (Hayiou-Thomas et al., 2006). Because not all measures were administered to each birth cohort, the numbers vary for specific analyses; the relevant sample sizes are included in the tables to be presented.

### 2.2 Measures of child language development

#### 2.2.1 Child language at 3 years (parent report)

This measure is a UK-adaptation of the CDI-III (Fenson et al., 2007) which included a 100-item checklist of expressive vocabulary, and a grammar scale consisting of a set of 12 sentence pairs for which the parent chose the member that most sounded like the child's current language. Examples are *That my* truck vs. *That's my* truck, and *Why he run* away vs. *Why did he run away?* Information on the validity of this measure and the language measure at 4 years is summarized in Dale, Price, Bishop, and Plomin (2003). The vocabulary and grammar scores were separately standardized and then averaged to yield a single score for language at 3 years, which was normally distributed with no skew.

#### 2.2.2 Child language at 4 years (parent report)

A new measure was developed specifically for this age in TEDS. The first part was a 48-item expressive vocabulary checklist. The second part asked the parents to indicate on a scale of 1–6 a global rating of the complexity of their child's language, from ‘not yet talking’ to ‘talking in long and complicated sentences’. The vocabulary and grammar scores were standardized and averaged to yield a single score for language at 4 years. This score showed negative skew of −.75.

#### 2.2.3 Child language at 4.5 years (restricted sample with in-home testing)

The verbal subtests of the battery were used to define a language composite; they included measures of oral vocabulary (McCarthy Scales of Children's Abilities Word Knowledge subtest), verbal fluency (MCSA Verbal Fluency), expressive semantics (Renfrew Bus Story Information score), expressive syntax (Refrew Action Picture Test Grammar score), receptive syntax (BAS Verbal Comprehension subtest), verbal memory for meaningful material (MSCA Verbal Memory Words and Sentences), Following the analysis of Hayiou-Thomas et al. (2006; see also Kovas et al., 2005), scores on these tests were standardized and averaged to yield a single score at 4.5 years. It should be noted that this sample was identified and recruited to oversample low performance; as a result, the sample was normally distributed, with little skew (0.11 in the present sample), but with mean scores .5–.75 SD below the mean for these tests.

### 2.3 Measures of parental language input style

Parents completed a questionnaire about their verbal interactions with each twin, when their children were 3 years old and again when they were 4. Questions about seven behaviors were utilized in the present analysis. Although the behaviors had not been selected on the basis of a comprehensive review of child-directed speech, each of them has been identified and studied as potentially relevant, positively or negatively, for language development. As shown below, parents first responded with respect to the elder twin, utilizing a five point scale (‘never or rarely’ to ‘almost always’). Then they were asked for each behavior, ‘Do you do this more or less with your 2nd born twin?’, responding on a five point scale (‘a lot more’ to ‘a lot less’). The score for the elder twin was the standardized response to the first question; the response for the younger twin was that score minus the standardized ‘more or less’ response (minus was appropriate to reverse the scoring direction of the second question). Following Asbury, Wachs, and Plomin (2005), affirmative responses to four of the questions were summed and standardized as reflecting a reported informal language stimulation approach (‘informal parent–child communication’ in Asbury et al.), and affirmative responses to three other questions were summed and standardized as reflecting a reported corrective feedback approach (‘instructive parent–child communication’ in Asbury et al.). The two patterns were not assumed to be mutually exclusive. Asbury et al. estimate internal consistency (alpha) of .50 for the language stimulation factor, and .86 for the corrective feedback factor. Informal language stimulation showed substantial negative skew at both 3 (−.6) and 4 (−.68) years; while corrective feedback was less skewed (.00 and .24 at the two ages, respectively), and showed a more nearly uniform distribution.

#### 2.3.1 Informal language stimulation

1. Does your 1st born twin take part in nursery rhymes, simple songs, or prayers?
2. Do you teach your 1st born twin about directions and locations (for example, left and right, where the shops are)?
3. Does your 1st born twin read books or look at books with you?
4. Do you talk to your 1st born twin when you are doing household chores?

#### 2.3.2 Corrective feedback

5. Do you ever correct words that your 1st born twin pronounces wrongly (for example, if s/he says ‘boon’ for ‘spoon’?
6. Do you ever correct your 1st born twin's sentence structure (for example, is s/he says ‘me not want it’ instead of ‘I don’t want it’?
7. Do you ever correct your 1st born twin if s/he says the wrong word for something (for example, if s/he calls a cow a horse)?

### 2.4 Data analysis

Because the shared age, and in two-thirds of cases the shared gender, within twin pairs might inflate twin correlations, age and sex were regressed out of all measures used for genetic analyses, as is typical of twin research. In addition, outliers more than 3 SD from the mean of each measure were removed.

Phenotypic analyses are reported first, including means by sex and zygosity, and correlations among the parental input and child language measures. Analyses of variance (ANOVAS) were conducted to assess the effects of sex and zygosity on the measures. In the genetic analyses, we first report intraclass twin correlations (ICCs) separately for each measure. These correlations can be used to make initial estimates of the role of genetic, shared environment, and nonshared environment influences (Plomin et al., 2013). MZ correlations greater than DZ correlations suggest a genetic effect (symbolized as *h*^2^ or *a*^2^, and which may be roughly estimated by doubling the difference), DZ correlations greater than half the MZ correlations suggest a shared environmental influence (symbolized as *c*^2^), and MZ correlations less than 1.0 suggest some influence of nonshared environment (symbolized as *e*^2^), as well as measurement error. More comprehensive and accurate measures of these influences are provided by standard univariate structural equation modeling, using specialized software (this study used OpenMx; Boker et al., 2011). Modeling allows the estimation of confidence intervals for three parameters described above, as well as comparing the fit of alternative models. Of particular importance for the present paper is whether there is significant genetic influence on the reported parental input measures, as this constitutes evidence for gene–environment correlations. Evidence for genetic effect on parental behavior such as language interaction style from a child-based twin study must reflect genetic differences between the twins. These analyses are followed by bivariate analyses of the concurrent and longitudinal correlations between parental input and child language measures (when those correlations are substantial). These analyses allow us to estimate the contribution of genetic, shared environment, and nonshared environment influences to those correlations. For example, the extent to which the correlation between two measures is higher for MZ twins than for DZ twins (these are cross-trait, cross-twin correlations, i.e., measure A for twin-1 is correlated with measure B for twin-2) is an index of the contribution of genetic factors that influence both measures in children. In the present study, this logic is applied to the correlation between parent input and child language measures. The outcome of these analyses are bivariate heritability, bivariate shared environmentality, and bivariate nonshared environmentality, which add to 1.0. The first of these is the most important for the present research questions. In addition, longitudinal phenotypic partial correlations provide additional evidence on possible causal relationships.

## 3 Results

### 3.1 Descriptive statistics for parent and child measures by gender and zygosity

Table 1 summarizes means and standard deviations of the standardized measures (*z*-scores), along with analysis of variance for sex and zygosity. The analyses are based on one randomly selected twin per family, to maintain independence of data. (The use of only half the sample explains why the mean is not precisely 0, as it would be if all participants were included.) Parent-reported language stimulation was very slightly higher, and use of corrective feedback very slightly lower in females. Although the large size of the sample renders these effects and those of zygosity significant, they are all very small, with the combined effect of sex and zygosity never exceeding 1.3% of the variance. For this reason, data from the two sexes are combined in the following analyses.

### 3.2 Do parent input measures predict child language?

Correlations among the reported language input and child language measures are presented in Table 2. These correlations are based on age- and sex-regressed measures. Child language shows moderate stability from 3 to 4.5 years, with age-to-age correlations exceeding .55. The two language input factors are also stable from 3 to 4, with correlations of .52 and .49, for language stimulation and corrective feedback respectively. Language stimulation is consistently and positively, though weakly, related to child language (*r* = .22–.27), whereas corrective feedback is negatively and even more weakly related (*r* = −.06 to −.02). All these correlations are statistically significant.

### 3.3 Is there evidence for gene–environment correlation?

Table 3 includes intraclass correlations (ICCs) and estimates of the genetic, shared environment, and nonshared environment influences for each of the reported parental input and child language variables, based on structural equations modeling. Standard tests of model fit, summarized in Table 4 (see table note for details), confirmed that in every case, the best-fitting model included significant effects for all three influences (ACE models). The language stimulation factor was approximately 30% heritable at both ages (i.e., genetic influences accounted for approximately 30% of the variance in this measure); corrective feedback was approximately 40% heritable. That is, these nominally environmental variables were significantly correlated with individual children's genetic makeup (the same for MZ twins; different for DZ twins). Consistent with earlier analyses of this dataset (Spinath et al., 2004; Hayiou-Thomas et al., 2012), the child language measures were significantly genetically influenced, with heritability figures in the .25–.40 range.

### 3.4 What proportion of the phenotypic correlations is due to shared genetic influences?

**Fig. 2:**
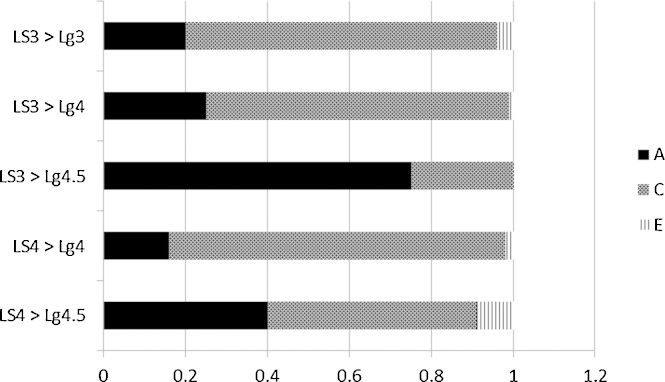
Proportion of the phenotypic correlation of parental language stimulation at 3 or 4 years with child language outcome measures which is due to genetic (black), shared environment (shaded), and non-shared environment (vertical lines) influences. LS = informal language stimulation; Lg = child language.

The third research question asks to what extent the gene–environment correlation identified above in fact influences the observed phenotypic correlations between reported parental input and child language. Bivariate analyses identify the proportion of the total phenotypic correlation that is due to common genetic influences (bivariate heritability), common shared environmental influences (bivariate shared environment), and common nonshared environmental influences (bivariate nonshared environment). These analyses were performed only for the informal language stimulation factor, as the cross-trait, cross-twin correlations (e.g. parent language input for twin 1 correlated with child language for twin 2) were significant only for that input factor. This is a criterion to assure reliable covariance for decomposition. The results of these analyses are reported in Table 5, and illustrated in Fig. 2. With respect to the prediction from age 3, approximately one-quarter of the prediction to the age 3 and 4 measures was due to bivariate heritability, and more than three-quarters of the prediction to the age 4.5 measures was due to bivariate heritability. The figures were similar, but somewhat lower with respect to the concurrent correlation between input at 4 and child language at 4 and 4.5.

### 3.5 Is there phenotypic evidence for directionality of effects?

We computed the prediction from 3 year reported language stimulation to 4 year language partialling out 3 year language as an index of the effect of language stimulation on child language, and compared it with the prediction from 3 year language to 4 year reported language stimulation partialling out 3 year language stimulation as an index of the effect of child language on language stimulation. Both correlations dropped substantially from the zero-order values in Table 2, reflecting the high stability from age 3 to 4 of both language stimulation (.52) and verbal ability (.65). Nevertheless, the partial correlations were still significant (both *p* < .001), and the correlation indexing the effect of language stimulation (.13) was significantly higher than the correlation indexing the effect of child language (.07) using the Fisher r-to-z transformation (*p* = .016, *df* = 4002 and 4078 respectively). Both the correlations and the difference are significant but small, suggesting influences in both directions, possibly larger from parent to child.

## 4 Discussion

The self-report measures of parental input used in this study are modestly but significantly related to child language outcomes. As expected, the informal language stimulation factor has a positive prediction to child language (.22–.27), while corrective feedback has a negative prediction (−.06 to −.12). Furthermore, the predictions for informal language stimulation are noticeably larger in absolute value. With respect to their reliability and validity, note that both measures were moderately stable (*r* about .5) from 3 to 4. The correlations from language stimulation to child language, while significant, are smaller than those in the literature which are based on direct observation (see Hoff, 2006, for a review). The language stimulation factor here was quite general, whereas most research in this area has examined more specific behaviors, such as sentence recasts and expansions; those specific factors may lead to higher predictions. It is also notable that the correlations from parental input quantity and richness of vocabulary, which may be closest to the present measure, tend to be smaller than other parent–child correlations in previous research (Hoff, 2006), and hence show the least discrepancy with the present results.

Both dimensions of reported parental language input have significant, moderate heritability, confirming gene–environment correlation. As noted earlier, these correlations can only be based on genetic differences between the twins. The significant heritability and shared environment parameters also provide a kind of concurrent validity for the input measures, as they can be interpreted as measuring the degree of relationship between the parental language input measures and genetic and shared environment factors, respectively. (Because nonshared environment also includes measurement error, it is not relevant for validity.)

A significant proportion of each of the phenotypic predictions from parental input to concurrent or later child language was due to shared genetic effects (.20–.73, median .40). Interestingly, both the univariate and bivariate heritabilities are higher for the prediction of the direct, 4.5 yr measure of child language than for the parent-reported measures. Shared environmental factors also played a significant and substantial role.

Because parents provided information on both their children's language and their own language input, there is a possibility of rater-bias (insufficient distinction between their own and their child's behavior) or insufficient distinction between their input to the two twins. If either or both of these processes were to occur, the correlations between the twins should be inflated for both MZ and DZ twins, which would inflate the estimates in bivariate shared environmental factors. Interestingly, the bivariate shared environmentality was quite high for the parent-reported child language measures at 3 and 4, but not for the in-person assessment at 4.5. Thus there may be inter-rater bias for the former, but not (or less) for the latter, which actually yields the stronger results with respect to our research questions.

Taken together, the heritability of the reported parental factors and the longitudinal bivariate results provide considerable evidence that genetic factors clearly play a role in the nature of parental input and its observed relationship with language development. However, it should also be noted that the phenotypic correlations are not entirely due to genetics; they are also in part due to shared environment influences on both parent and child. These results should be regarded as initial estimates, given the likely limited validity of the input variables with respect to distinguishing the twins and accuracy of self-report of behavior. Direct measures of the input would be highly preferable, but much more resource-intensive, especially because they must be child-specific, not family-general, effectively doubling the effort.

Can we distinguish passive from evocative gene–environment (GE) correlation with the present data? No, the classical twin study cannot do this; other designs are needed, e.g., adoption studies, or children of twins designs (Plomin et al., 2013). Some suggestive evidence that there is both evocative GE correlation along with stronger passive GE correlation comes from the phenotypic partial correlations, which are both significant, and significantly – but only slightly – stronger from parent to child than conversely. The negative results from DeThorne and Hart (2009), which focused on evocative GE correlation is also consistent with this impression. However, stronger genetically sensitive designs, such as adoption studies and the children of twins designs, will be needed for more definite conclusions on this question.

A somewhat unexpected aspect of the results was the substantial heritability of the reported corrective feedback factor, indeed higher than that for language stimulation, combined with a very weak prediction to child language. The heritability of corrective feedback means that it reflects genetic influences in the child. However, these are not necessarily genetic influences specifically on language. For example, they might be related to behavioral issues which evoke an orientation toward behavior management, which ‘spills over’ into correcting speech and language. Alternatively, parental ‘teacherly style with concern for correct form’ might reflect a trait observable in the parent, which emerges only later in the development of the child, perhaps in the form of more advanced metalinguistic awareness. Research which compares parental language interaction style with other aspects of parental behavior is needed to clarify this result.

### 4.1 Limitations

The most important limitation to the conclusions of this study stems from the use of parent-report measures of language input, rather than some form of direct observation. As this measure was innovative to the present study, there is no independent evidence concerning its reliability and validity. Above we have provided some evidence that these measures have significant validity. In addition, they are highly global measures rather than more specifically focused measures suggested by the current literature, such as vocabulary diversity or proportion of sentence recasts. The low internal consistency of informal language stimulation could also be viewed as a limitation. Detailed examination of correlations revealed that all four component questions for informal language stimulation related approximately equally to the composite, and approximately equally to the language outcome measures. This pattern suggests that informal language stimulation may not be unidimensional, but that all components contribute to the overall prediction.

In addition, the exact wording of the self-report questions was not ideal for the present purposes. The basic question frame of asking about one twin first, and then about the difference between the twins (rather than about the second twin directly) may have inflated differences between the twins. If this effect occurred equally for both MZ and DZ twins, the estimate of genetic influence on parental behavior would have been spuriously lowered and the effect of nonshared environment increased. Alternatively, if responses for MZ twins were more affected (because it led the parents to search for differences that were in fact minimal) than for DZ twins, the estimate of genetic influence would again have been diminished, and that for shared environment increased. Both cases thus would yield an underestimate of genetic influence. A final issue for the use of self-report is that parent responses to questions #1 and #3 may have reflected individual differences in children as well as the parents’ own behavior, potentially inflating the correlation between parent and child.

Another limitation of the design is that the youngest age at which the environmental measure was available in TEDS was 3 years. It is possible that aspects of parental input are even more important at younger ages when it forms an even larger proportion of the total input and the need for joint attention is especially critical.

## 5 Conclusion

Genetic research of this type is easily misunderstood. The broadest conclusion from this work is not genetic determinism. Genes need the environment to have their effect. A child with a strong genetic endowment for mathematics, or for music, or for athletics does not became exceptionally skilled without years of practice; the genes may have their largest effect by inclining the child to spend his or her time in that practice (cf. Detterman, 2014, and associated papers for a discussion of practice, ability, and expertise). Similarly, the genes shared by parent and child lead to forms of ‘language practice’ which, in their quantity and their quantity, facilitate child language development. This genotype-environment correlation in no way precludes the effectiveness of interventions for parents or for children, and many interventions have been developed with both short-term and long-term effects (Finestack & Fey, 2013; Girolametto et al., 2013). Nevertheless, we suggest that it is important to understand that correlations between parent language and child language development are not always causal; they often involve correlations between genetic propensities of parents and their children, a conclusion which has important implications for intervention and prevention as well as interpretation. Awareness of the ways in which genes and environment interplay are essential for developing better interventions, for example, in understanding the bidirectional transactions between parent language and child language development and the genotype-environment feedback loops. It has often been pointed out that genetically sensitive designs such as twin studies can provide the strongest possible evidence for environmental effects. For example, Hardy-Brown, Plomin, and DeFries (1981) concluded from their adoption study that maternal responsiveness to infant vocalizations played an important, purely environmental role in making the auditory speech channel salient to infants, and therefore it would be a good focus for intervention. Beyond such specific lessons, research on gene–environment correlations provides a lesson in respecting the individuality each person – parent and child – brings to the language learning context.

## Figures and Tables

**Table 1:** Standardized parental input measures at 3 years, and child language measures at 3, 4, and 4.5 years.

	Measures	Means and standard deviation on standardized data										ANOVA-effects of sex and zygosity						
		All		Females		Males		MZ		DZ		Sex		Zygosity		Sex * zyg.		
		*M* (*N*)	SD	*M* (*N*)	SD	*M* (*N*)	SD	*M* (*N*)	SD	*M* (*N*)	SD	*p*	*η*^2^	*p*	*η*^2^	*p*	*η*^2^	*R*^2^
1	Informal language stimulation at age 3	0.03 (*N* = 5465)	0.91	0.13 (*N* = 2778)	0.87	−0.08 (*N* = 2687)	0.94	−0.04 (*N* = 1857)	0.89	0.06 (*N* = 3608)	0.92	0.00	0.01	0.00	0.00	0.10	0.01	0.000
2	Corrective feedback age 3	0.02 (*N* = 5501)	0.97	−0.05 (*N* = 2783)	0.99	0.08 (*N* = 2718)	0.95	0.08 (*N* = 1881)	0.93	−0.02 (*N* = 3620)	0.99	0.00	0.03	0.00	0.03	0.09	0.00	0.000
3	Child language age 3	0.00 (*N* = 5065)	1.00	0.01 (*N* = 2584)	0.98	−0.01 (*N* = 2481)	1.02	−0.08 (*N* = 1712)	1.04	0.04 (*N* = 3353)	0.97	0.17	0.00	0.00	0.04	0.21	0.00	0.000
4	Informal language stimulation at age 4	0.03 (*N* = 7277)	0.93	0.12 (*N* = 3733)	0.91	−0.07 (*N* = 3544)	0.94	−0.02 (*N* = 2445)	090	0.05 (*N* = 4832)	0.94	0.00	0.01	0.00	0.00	0.07	0.00	0.013
5	Corrective feedback age 4	0.01 (*N* = 7319)	0.98	−0.04 (*N* = 3740)	1.03	0.06 (*N* = 3579)	0.96	0.07 (*N* = 2466)	0.95	−0.03 (*N* = 4853)	1.00	0.00	0.00	0.00	0.00	0.16	0.00	0.005
6	Child language age 4	0.00 (*N* = 4373)	1.00	0.00 (*N* = 2220)	.096	0.00 (*N* = 2153)	1.03	−0.08 (*N* = 1481)	1.05	0.05 (*N* = 2892)	0.96	0.95	0.00	0.00	0.00	0.49	0.00	0.003
7	Child language at age 4.5	0.00 (*N* = 805)	0.99	−0.01 (*N* = 370)	1.05	0.02 (*N* = 435)	0.94	−0.14 (*N* = 286)	0.96	0.09 (*N* = 519)	1.00	0.43	0.00	0.00	0.01	0.21	0.00	0.011

*N* = sample size based on one randomly selected twin in the pair; *M* = mean; SD = standard deviation; *p* = *p*-value of the effects of sex on variables; *η*^2^ = eta-squared and cleared of outliers scores (±3 standard deviations). *R*^2^ = variance explained by sex and zygosity.

**Table 2:** Correlations between parental input measures and child language outcomes, and stability correlations.

		1	2	3	4	5	6	9
1	Informal language stimulation at age 3	1						
	*N*	5465						
2	Corrective feedback age 3	.06**	1					
	*N*	5438	5501					
3	Child language age 3	.27**	−.09**	1				
	*N*	5011	5043	5065				
4	Informal language stimulation at age 4	.52**	.04*	.21**	1			
	*N*	4410	4439	4118	7277			
5	Corrective feedback age 4	.03	.49**	−.08**	.07**	1		
	*N*	4431	4468	4138	7227	7319		
6	Child language age 4	.27**	−.06**	.65**	.24**	−.07**	1	
	*N*	4324	4355	4047	4275	4297	4373	
9	In-home language on whole sample	.25**	−.12**	.56**	.22**	−.13**	.58**	1
	*N*	495	504	452	784	796	470	805

Variables corrected for age and sex, outliers ±3 standard deviations excluded.

** Correlation is significant at the 0.01 level.

* Correlation is significant at the 0.05 level (2-tailed).

**Table 3:** Univariate genetic analyses of parental input and child language measures.

Measures		Intraclass correlations		Parameter estimates from best fitting univariate model		
		MZ	DZ	*a*^2^ (95% CI)	*c*^2^ (95% CI)	*e*^2^ (95% CI)
		ICC (95% CI) (*N*)	ICC (95% CI) (*N*)			
1	Informal language stimulation at age 3	0.85 (0.84; 0.86) (*N* = 1850)	0.70 (0.69; 0.72) (*N* = 3583)	0.32 (0.28; 0.35)	0.55 (0.52; 0.58)	0.13 (0.13; 0.14)
2	Corrective feedback age 3	0.84 (0.83; 0.86) (*N* = 1877)	0.65 (0.63; 0.67) (*N* = 3600)	0.45 (0.41; 0.49)	0.41 (0.38; 0.45)	0.14 (0.13; 0.15)
3	Child language age 3	0.91 (0.90; 0.92) (*N* = 1634)	0.74 (0.73; 0.66) (*N* = 3180)	0.30 (0.27; 0.33)	0.60 (0.57; 0.63)	0.10 (0.09; 0.11)
4	Informal language stimulation at age 4	0.86 (0.85; 0.87) (*N* = 2429)	0.70 (0.69; 0.72) (*N* = 4785)	0.32 (0.30; 0.35)	0.55 (0.52; 0.57)	0.13 (0.12; 0.14)
5	Corrective feedback age 4	0.85 (0.84; 0.86) (*N* = 2457)	0.67 (0.65; 0.68) (*N* = 4816)	0.40 (0.37; 0.43)	0.46 (0.43; 0.49)	0.14 (0.13; 0.15)
6	Child language age 4	0.88 (0.87; 0.89) (*N* = 1441)	0.71 (0.69; 0.73) (*N* = 2809)	0.27 (0.24; 0.31)	0.59 (0.56; 0.62)	0.14 (0.13; 0.15)
7	Child language at age 4.5	0.77 (0.70; 0.80) (*N* = 285)	0.54 (0.48; 0.60) (*N* = 514)	0.44 (0.31; 0.57)	0.33 (0.20; 0.44)	0.23 (0.20; 0.28)

Variables corrected for age and sex – outliers ±3 st. dev. removed.

**Table 4:** Summary of model fit.

Univariate model fit								
Measure	Model	−2LL	df	(Δ − 2LL)	AIC	BIC	*p*-Value	ep
Informal language stimulation at age 3	Saturated	24,211.33	10,933	–	2345.33	−74,645.29	–	10
ACE	24,239.48	10,939	28.14	2361.48	−74,671.40	0.00	4

Corrective feedback age 3	Saturated	26,205.19	10,996	–	4213.19	−73,221.09	–	10
ACE	26,250.95	11,002	45.76	4246.95	−73,229.58	.000	4

Child language at age 3	Saturated	23,313.55	10,117	–	3079.55	−68,164.77	–	10
ACE	23,348.70	10,123	35.15	3102.70	−68,183.88	0.00	4

Informal language stimulation at age 4	Saturated	32,545.06	14,532	–	3481.06	−98,853.87	–	10
ACE	32,563.67	14,538	18.61	3487.67	−98,889.51	0.00	4

Corrective feedback at age 4	Saturated	34,805.25	14,628	–	5549.25	−97,461.71	–	10
ACE	34,848.49	14,634	43.24	5580.49	−97,472.72	0.00	4

Child language at age 4	Saturated	20,520.91	8734	–	3052.91	−58,452.27	–	10
ACE	20,560.82	8740	39.91	3080.82	−58,466.61	0.00	4

Child language at age 4.5	Saturated	4134.49	1603	–	928.49	−10,359.90	–	10
ACE	4147.71	1609	13.23	929.71	−10,400.93	0.04	4

−2LL = −2 Log likelihood; df = degrees of freedom; −2LL = −2Log likelihood; Δ − 2LL = difference in likelihood between the compared models; AIC = Akaike Information Criterion; BIC = Bayesian Information Criterion. Smaller values of BIC and AIC index better fit; *p*-value = refers to significant drop in likelihood value between the Saturated and compared nested model; ep = estimated parameters.

Fit comparison between the saturated model, from observed data, and the best fitting genetic ACE model. In the ACE model the parameters are estimated as the effects of genetic (A), shared-environmental (C), and non-shared environmental (E) factors. The drop in likelihood between the saturated and full ACE model is significant in all 7 comparisons. However, in large samples the BIC index is considered more reliable than AIC in evaluating the fit relative to parsimony, as BIC takes into account the sample size. The BIC indicates a better fit here for the ACE models, as the index is consistently smaller (more negative) for those models.

**Table 5:** Bivariate analyses of the phenotypic correlations from parental input to child language.

Measures	Phenotypic correlations	Bivariate parameters		
		*a*^2^ (95% CI)	*c*^2^ (95% CI)	*e*^2^ (95% CI)
Informal language stimulation at age 3 with child language 3	0.27	**0.20 (0.12; 0.28)**	**0.76 (0.69; 0.83)**	**0.04 (0.01; 0.06)**
Informal language stimulation at age 3 with child language 4	0.27	**0.25 (0.16; 0.35)**	**0.74 (.65; 0 .81)**	0.01 (−0.01; 0.04)
Informal language stimulation at age 3 with child language 4.5	0.25	**0.73 (.29; 1.0)**	0.18 (−0.35; 0.57)	0.09 (−0.02; 0.21)
Informal language stimulation at age 4 with child language 4	0.24	**0.40 (.07; 0 77)**	**0.51 (0.19; 0.78)**	0.09 (−0.01; 0.19)
Informal language stimulation at 4 with child language 4.5	0.22	**0.47 (0.13; 0.85)**	**0.42 (0.07; 0.71)**	**0.11 (0.00; 0.21)**

Figures in bold identify significant effects.

## Footnotes

1 MZ twins are identical in terms of DNA sequence variation (at least as much as our DNA sequence at one age is identical to our DNA sequence at another age), whereas DZ twins are only 50% similar on average for DZ variants that operate in an additive manner. Epigenetic and other effects on gene expression may also add variability but are conceptualized within behavioral genetic research as environmental effects, a view which is supported by many recent findings of epigenetic influences such as methylation resulting from the organism's recent environmental experience.
